# Electrophysiological Response in Hypertensive Crisis: An Evaluation of the ICEB Score

**DOI:** 10.3390/medicina62030501

**Published:** 2026-03-09

**Authors:** Süleyman Kırık, Mehmet Göktuğ Efgan, Efe Kanter, Ecem Ermete Güler, Tutku Duman Şahan, Hanife Kübra Gezer

**Affiliations:** 1 Department of Emergency Medicine, Faculty of Medicine, Izmir Katip Çelebi University, Izmir 35100, Turkey; goktugefgan@gmail.com (M.G.E.); efekanter@hotmail.com (E.K.); ecemermete@hotmail.com (E.E.G.); karakurtkubra1996@gmail.com (H.K.G.); 2 Department of Emergency Medicine, Izmir Çeşme Alper Çizgenakat State Hospital, Izmir 35950, Turkey; tutkuduman75@gmail.com

**Keywords:** hypertensive crisis, ICEB score, electrophysiological balance

## Abstract

*Background and Objectives*: This study aimed to evaluate the pre- and post-treatment changes in ICEB and ICEBc score markers of cardiac electrical balance in patients presenting to the emergency department with hypertensive urgency and to investigate their relationship with short-term clinical outcomes. *Materials and Methods*: In this retrospective study, 50 patients who presented to a tertiary university hospital emergency department between 1 January 2021 and 31 December 2024 with a diagnosis of hypertensive urgency and had pre- and post-treatment 12-lead ECGs were analysed. ICEB (QT/QRS) and ICEBc (QTc/QRS) scores were calculated manually. Patients were categorized as discharged or hospitalized. Within-group and between-group comparisons of the scores were performed. *Results*: Of the patients, 66% were female, and the mean age was 60.4 ± 14.5 years. A statistically significant increase in ICEB scores was observed after treatment in discharged patients (*p* = 0.006), whereas ICEBc scores showed no significant change. In the hospitalized group, no significant difference was found in either ICEB or ICEBc scores. Additionally, between-group comparisons revealed no significant differences in ICEBc values. *Conclusions*: The ICEB score may serve as a dynamic marker reflecting the electrophysiological response to antihypertensive treatment. The observed increase in ICEB after treatment may indicate the restoration of electrical stability. In contrast, ICEBc appeared to have limited predictive value in this clinical context. Further prospective studies with larger populations are needed to determine the clinical utility of ICEB in the management of hypertensive crises.

## 1. Introduction

Hypertensive urgency is defined as a clinical condition characterized by a severe elevation in blood pressure, specifically a systolic blood pressure ≥ 180 mmHg or diastolic blood pressure ≥ 110 mmHg, without evidence of acute target organ damage [1,2]. This condition is a frequent cause of emergency department (ED) visits, typically presenting with distressing symptoms such as headache, palpitations, chest pain, anxiety, or blurred vision [3].

The primary management goal in these cases is twofold: to alleviate symptoms and to reduce blood pressure in a controlled manner. Treatment focuses on relieving the discomfort associated with acute elevations, such as anxiety and palpitations, while ensuring a gradual reduction in blood pressure to prevent end-organ hypoperfusion [4]. However, beyond immediate hemodynamic stabilization, it is crucial to recognize that sudden blood pressure surges can acutely stress the cardiovascular system, particularly affecting myocardial electrical activity [5]. Consequently, evaluating the cardiac electrophysiological response during treatment is of significant clinical importance.

Acute blood pressure surges influence myocardial electrophysiology through mechanisms such as increased afterload and autonomic imbalance [6]. These changes can alter ventricular depolarization and repolarization dynamics, potentially modulating the risk of arrhythmia. While traditional electrocardiographic markers like the QT interval and corrected QT (QTc) reflect repolarization, and the QRS duration reflects conduction, relying on either parameter in isolation may fail to fully capture the integrated electrical balance necessary to predict malignant ventricular arrhythmias [7].

To address this limitation, the Corrected Index of Cardiac Electrophysiological Balance (ICEBc) has emerged as a novel electrocardiographic parameter. Calculated as the ratio of the QTc interval to the QRS duration, ICEBc provides a more comprehensive view of cardiac electrical stability [8,9]. It has gained increasing attention for its potential to predict ventricular arrhythmias and sudden cardiac events across various clinical settings. Notably, elevated ICEBc scores have been associated with a higher risk of all-cause mortality and have been observed in conditions such as subarachnoid hemorrhage and white coat hypertension [10,11].

Given these associations, this study aims to evaluate pre- and post-treatment changes in ICEB and ICEBc in patients presenting to the ED with hypertensive urgency. Furthermore, we explore whether these electrophysiological changes differ according to short-term clinical disposition, specifically comparing patients discharged home versus those requiring hospitalization.

## 2. Materials and Methods

### 2.1. Study Design

This study was designed as a retrospective observational investigation to evaluate cardiac electrophysiological changes in patients with hypertensive urgency. The research was conducted at the Department of Emergency Medicine, İzmir Katip Çelebi University Atatürk Training and Research Hospital (İzmir, Türkiye). This facility serves as a tertiary-level academic center providing comprehensive 24 h emergency and cardiovascular care to a high-volume urban population. The study period spanned four years, including all eligible patient presentations between 1 January 2021 and 31 December 2024.

### 2.2. Ethical Considerations

The study was conducted in strict accordance with the guidelines of the Declaration of Helsinki. The protocol was reviewed and approved by the İzmir Katip Çelebi University Clinical Research Ethics Committee (Approval Date: 8 April 2025; Approval Number: 0206). Due to the retrospective nature of the analysis, the requirement for informed consent was waived by the ethics committee, as patient data were anonymized prior to analysis to ensure confidentiality.

### 2.3. Study Population and Inclusion Criteria

The study population consisted of patients aged 18 years and older who presented to the emergency department (ED) with a diagnosis of hypertensive urgency. For the purpose of this study, hypertensive urgency was defined according to current guidelines as a systolic blood pressure (SBP) ≥ 180 mmHg and/or a diastolic blood pressure (DBP) ≥ 110 mmHg, without clinical or laboratory evidence of acute target organ damage (TOD) [12].

Patients were included if they met the blood pressure criteria and had available high-quality 12-lead electrocardiograms (ECGs) recorded at two distinct time points:

Baseline: At presentation to the ED prior to medical intervention.
Post-treatment: After the administration of antihypertensive therapy and stabilization of blood pressure.

### 2.4. Exclusion Criteria

To ensure that the observed electrophysiological changes were attributable to blood pressure modulation rather than intrinsic cardiac pathology or external confounders, specific exclusion criteria were applied. Patients were excluded if they presented with evidence of acute target organ damage—such as hypertensive emergency, acute heart failure, or aortic dissection—or concurrent acute coronary syndromes and cerebrovascular events. Furthermore, the study excluded patients with permanent cardiac pacemakers or implantable cardioverter-defibrillators (ICD), as well as those with persistent arrhythmias, such as chronic atrial fibrillation, that preclude accurate QT measurement. Finally, patients exhibiting significant electrolyte imbalances (e.g., hypokalemia, hypocalcemia) on admission, those with incomplete or technically inadequate ECG tracings, and women who were pregnant or lactating were not included in the analysis.

### 2.5. Clinical Protocols and Data Collection

Demographic data (age, sex) and clinical characteristics were extracted from electronic medical records. Patients diagnosed with hypertensive urgency were identified based on elevated blood pressure values without evidence of acute target organ damage. Patients who received intravenous antihypertensive therapy were excluded to ensure a homogeneous study cohort reflecting “urgency” rather than “emergency” management.

The initial electrocardiogram (ECG) was obtained at the time of admission to the emergency department, prior to the initiation of antihypertensive treatment.

Antihypertensive management in the ED followed standard departmental protocols, primarily utilizing short-acting oral agents such as captopril (ACE inhibitor) or amlodipine (calcium channel blocker). In a minority of cases, alternative oral agents were administered based on clinical judgment.

After treatment and clinical reassessment, patients were either discharged directly from the ED or admitted to the hospital according to their clinical status, blood pressure response, and the presence of suspected target organ involvement. The grouping in the study reflects this disposition decision rather than in-hospital outcomes.

Post-treatment ECGs were obtained approximately 60–90 min after the administration of oral medication, once blood pressure control had been achieved, allowing for stabilization of transient hemodynamic fluctuations.

Patients were stratified into two subgroups based on their final ED disposition:

Discharged Group:

Patients who achieved symptom relief and blood pressure targets and were discharged home. Hospitalized Group: Patients who were admitted to the ward for further monitoring or treatment. This stratification served as a proxy for clinical severity to investigate whether the electrophysiological response differed between routine cases and those requiring extended care.

### 2.6. Electrocardiographic Measurements

Standard 12-lead ECGs were recorded at a paper speed of 25 mm/s and a voltage of 10 mm/mV. All ECG parameters were measured manually. The QT interval was measured from the onset of the QRS complex to the end of the T wave, while the QRS duration was measured from the onset of the Q wave (or R wave if no Q wave was present) to the end of the S wave. To correct for heart rate, the corrected QT interval (QTc) was calculated using Bazett’s formula, defined as the QT interval divided by the square root of the RR interval. Subsequently, the Index of Cardiac Electrophysiological Balance (ICEB) was determined by dividing the QT interval by the QRS duration, and its corrected form (ICEBc) was calculated as the ratio of the QTc interval to the QRS duration. Measurements were performed independently by two researchers blinded to the patient’s clinical outcome. To ensure reliability, inter-observer agreement was assessed using Cohen’s kappa (κ) coefficient, and in cases of significant disagreement (>20 ms difference), a third senior emergency medicine specialist adjudicated the measurement to reach a consensus.

### 2.7. Statistical Analysis

Data analysis was performed using IBM SPSS Statistics for Windows, Version 27.0 (IBM Corp., Armonk, NY, USA). Continuous variables were evaluated for normality using the Kolmogorov–Smirnov test combined with visual inspection of histograms. Based on the distribution characteristics, continuous data are presented as mean ± standard deviation (SD) for normally distributed variables or median with minimum–maximum range for non-normally distributed data, whereas categorical variables are summarized as frequencies (n) and percentages (%). To evaluate the changes in ICEB and ICEBc, a mixed-design analysis of variance (ANOVA) was conducted; this model included “Time” (pre- vs. post-treatment) as the within-subjects factor and “Disposition” (Discharged vs. Hospitalized) as the between-subjects factor. Effect sizes were quantified using partial eta squared (ηp^2^) reported alongside F-statistics, and a two-tailed *p*-value of <0.05 was considered statistically significant for all analyses.

## 3. Results

A total of 50 patients were included in the study, of whom 33 (66%) were female. The mean age was calculated as 60.40 ± 14.53 years. While 43 patients (86%) were discharged from the emergency department, 7 patients (14%) were admitted for further inpatient care. Descriptive statistics related to the study population are presented in Table 1.

As shown in Table 2, in accordance with the results, within-group comparisons revealed a significant increase in ICEB values after treatment compared to baseline in patients who were discharged (F = 8.364; *p* = 0.006; η^2^ = 0.148). In contrast, no significant change in ICEB values was observed among patients who were hospitalized (F = 0.809; *p* = 0.373; η^2^ = 0.017).

**Figure 1:**
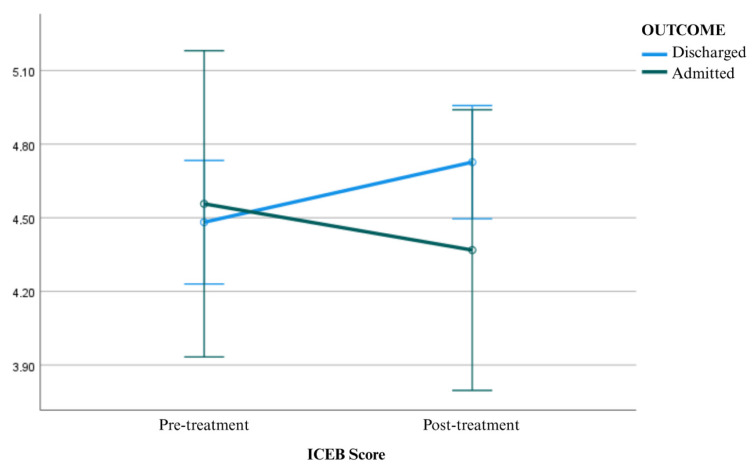
Mean ICEB scores before and after antihypertensive treatment, with error bars representing the standard error of the mean (SEM).

There was no statistically significant difference in pre-treatment ICEB values between discharged patients and those who were hospitalized (F = 0.050; *p* = 0.823; η^2^ = 0.001). Similarly, no significant difference was detected between the two groups in terms of post-treatment ICEB values (F = 1.365; *p* = 0.248; η^2^ = 0.028). Comparison of ICEB measurements before and after antihypertensive treatment in patients presenting with hypertensive urgency is presented in Table 2, and graphical representations of mean ICEB scores before and after antihypertensive treatment are presented in Figure 1.

**Figure 2:**
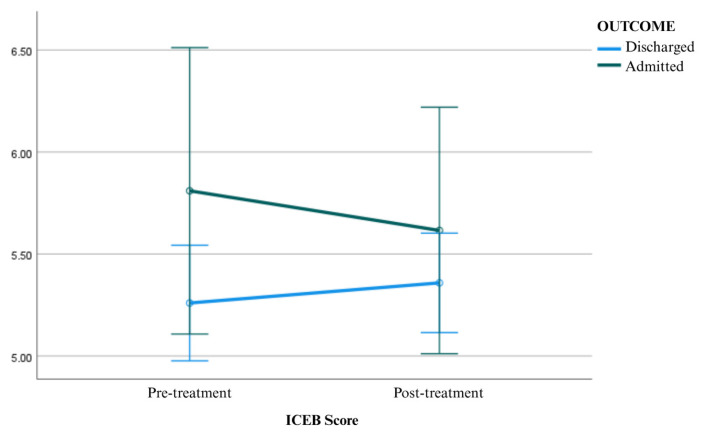
Mean ICEBc scores before and after antihypertensive treatment, with error bars representing the standard error of the mean (SEM).

As shown in Table 3, in accordance with the results, there was no statistically significant difference in pre-treatment ICEBc values between discharged patients and those who were hospitalized (F = 2.133; *p* = 0.151; η^2^ = 0.043). Similarly, no significant difference was found between the two groups in terms of post-treatment ICEBc values (F = 0.630; *p* = 0.431; η^2^ = 0.013). In within-group comparisons, no significant change was observed in ICEBc values before and after treatment in either the discharged group (F = 1.148; *p* = 0.289; η^2^ = 0.023) or the hospitalized group (F = 0.725; *p* = 0.399; η^2^ = 0.015). Graphical representations of mean ICEBc scores before and after antihypertensive treatment are presented in Figure 2.

## 4. Discussion

The primary objective of this study was to evaluate the dynamic changes in cardiac electrophysiological balance, measured by ICEB and ICEBc, in patients treated for hypertensive urgency. Our most significant finding is that the raw ICEB score significantly increased following treatment in patients who were clinically stable enough to be discharged, whereas no such recovery was observed in patients requiring hospitalization. Conversely, the rate-corrected index (ICEBc) did not demonstrate statistically significant changes. These results suggest that ICEB may serve as a more sensitive marker of acute electrophysiological recovery than ICEBc in the setting of sudden hemodynamic stress.

The concept of the Index of Cardiac Electrophysiological Balance (ICEB), defined as the ratio of the QT interval to the QRS duration, was introduced to quantify the equilibrium between ventricular repolarization and depolarization [8]. While traditional markers such as QT or QTc intervals are well-established predictors of arrhythmia [12], they reflect repolarization in isolation. ICEB offers a distinct advantage by integrating conduction velocity (QRS) and repolarization (QT) into a single index, theoretically providing a more holistic view of myocardial electrical stability [13,14]. Previous research has established that deviations in ICEB—either significant elevations or reductions—are associated with increased susceptibility to malignant arrhythmias, including Torsades de Pointes and ventricular tachycardia [10,15].

In our cohort, the significant post-treatment rise in ICEB among discharged patients likely reflects the restoration of physiological balance. Physiologically, acute hypertensive episodes are characterized by a surge in sympathetic tone, increased ventricular afterload, and subclinical myocardial ischemia [16,17]. These stressors can disrupt membrane stability, often resulting in a transient suppression of the QT interval or prolongation of the QRS complex, effectively lowering the ICEB ratio [18]. The administration of antihypertensive therapy alleviates ventricular wall stress and reduces the autonomic burden [19,20,21,22,23]. Consequently, the increase in ICEB observed in the discharged group suggests a normalization process—a “rebound” toward stability following the resolution of acute stress. This aligns with recent findings by Polatkan et al., who observed a significant post-procedural rise in ICEB (4.1 to 4.5) in patients undergoing atrial septal defect closure, supporting the utility of ICEB as a dynamic marker of hemodynamic relief [16].

The divergence in outcomes between the discharged and hospitalized groups is of particular clinical interest. While discharged patients demonstrated measurable electrophysiological recovery, the hospitalized group showed no significant change in ICEB despite blood pressure management. This persistent electrophysiological inertia may indicate underlying subclinical instability or a higher burden of cardiovascular disease that prompted the clinical decision to admit. Therefore, a lack of ICEB improvement after initial treatment could potentially serve as a subtle warning sign, identifying a subgroup of patients who—despite improved blood pressure numbers—remain at higher risk for adverse events.

Interestingly, while ICEB proved responsive to treatment, the corrected index (ICEBc) did not. Theoretically, ICEBc should be superior because it accounts for heart rate variability. However, in the context of the emergency department, where patients experience rapid fluctuations in heart rate due to pain, anxiety, and autonomic discharge, the reliability of standard correction formulas (e.g., Bazett’s) is frequently challenged. Sudden shifts in sympathetic activity can alter the QT interval and heart rate non-linearly, potentially rendering the mathematically derived ICEBc less sensitive to acute changes than the raw ICEB [9,15]. This may explain why ICEBc failed to capture the recovery patterns observed with the uncorrected index in this specific acute setting. Although elevated ICEBc levels have been associated with adverse outcomes in certain pathological states, the interpretation of ICEB should be context-dependent. In our study, ICEB changes were evaluated in the acute setting of hypertensive urgency and reflect short-term electrophysiological modulation following blood pressure reduction rather than long-term prognostic risk. Therefore, transient increases or decreases in ICEB should not be directly equated with improved or worsened clinical outcomes.

It is also important to address the potential confounding effect of pharmacotherapy. The treatment protocol in our study primarily utilized short-acting oral agents, such as captopril and amlodipine, without the use of intravenous antihypertensives [24]. Because these agents were chosen for their hemodynamic efficacy rather than direct antiarrhythmic properties, and because no intravenous drugs were used, the observed ECG changes are most plausibly attributed to the physiological benefits of blood pressure control rather than direct drug-induced ion channel modulation.

### 4.1. Clinical Implications and Future Directions

From a practical standpoint, ICEB is a highly attractive biomarker for the emergency setting. It is non-invasive, cost-effective, and easily derived from a standard 12-lead ECG without requiring specialized software [18]. Our findings suggest that calculating the delta-ICEB (pre- vs. post-treatment) could provide an additional layer of safety data when considering a patient for discharge. A rise in ICEB may corroborate clinical stability, while a static ICEB might prompt prolonged observation.

However, these results must be interpreted in light of certain limitations. This was a single-center, retrospective study, and while we adjusted for disposition, we could not control for all long-term comorbidities. Furthermore, data regarding ICEB dynamics in hypertensive crises are scarce, meaning our results represent a novel contribution that requires validation in larger, prospective, multi-center cohorts. Future studies should ideally correlate these acute electrical changes with long-term adverse cardiovascular events to definitively establish the prognostic value of ICEB in hypertensive urgency.

### 4.2. Limitations

This study has several limitations. First, its retrospective design limits the ability to establish causal relationships. Additionally, the relatively small sample size, particularly the hospitalized subgroup (n = 7), may reduce the statistical power and restrict the generalizability of the findings. Only short-term ECG data were evaluated, and long-term arrhythmic outcomes were not assessed; therefore, the clinical relevance of the observed ICEB changes for hard endpoints remains uncertain. Although ECG measurements were performed manually with a consensus approach, residual measurement variability is possible, and ICEBc may be influenced by the QT correction method used under conditions of acute heart rate and autonomic fluctuations. Finally, laboratory and clinical covariates that could affect repolarization may not have been fully captured in a retrospective dataset. Echocardiographic data and systematic arrhythmia monitoring were not available for all patients due to the retrospective design of the study, which may limit the ability to correlate ICEB changes with structural or clinical arrhythmic outcomes.

## 5. Conclusions

In this retrospective cohort of hypertensive urgency patients, ICEB showed significant short-term changes post-antihypertensive treatment, especially in emergency department discharged patients, while ICEBc did not. In emergency situations, ICEB may indicate acute electrophysiological modulation associated with blood pressure reduction and provide additional information beyond standard ECG parameters. However, the retrospective design, single-center context, and small sample size warrant caution. More extensive, prospective, and longitudinal studies are needed to determine ICEB dynamics’ clinical significance and their potential role in hypertensive crisis risk stratification and outcome evaluation.

## Figures and Tables

**Table 1:** Descriptive Statistics of the Study Population.

	Statistics
Age *	60.40 ± 14.53 (95% CI: 56.3–64.5)
**Gender**, *n* (%)	
Female	33 (66)
Male	17 (34)
Pre-treatment systolic BP *	183.10 ± 14.61 (SEM = 2.07)
Pre-treatment diastolic BP *	95.96 ± 18.06 (SEM = 2.56)
Post-treatment systolic BP *	136.68 ± 9.51 (SEM = 1.34)
Post-treatment diastolic BP *	74.94 ± 9.70 (SEM = 1.37)
Pre-treatment ICEB Score *	4.67 ± 0.75 (95% CI: 4.45–4.89)
Post-treatment ICEB Score *	4.49 ± 0.81 (95% CI: 4.26–4.72)
**Outcome**, *n* (%)	
Discharged	48 (86)
Admitted	7 (14)

Values *n*: Number of patients, %: Sample percentage. *: Presented as *mean ± standard deviation* and *median (min-max).*

**Table 2:** Comparison of ICEB Measurements Before and After Antihypertensive Treatment in Patients Presenting with Hypertensive Urgency: Discharged vs. Hospitalized Patients.

	Subgroups		Test Statistics ^†^		
	Discharged	Admitted	*F*	*p*	*η* ^2^
**ICEB**					
Pre-treatment	4.48 ± 0.82	4.55 ± 0.77	0.050	0.823	0.001
Post-treatment	4.72 ± 0.75	4.36 ± 0.71	1.365	0.248	0.028
**Test Statistics ^¥^**	***F* = 8.364; *p* = 0.006; *η*^2^ = 0.148**	*F* = 0.809; *p* = 0.373; *η*^2^ = 0.017			

F: Mixed Design ANOVA; Effect Size (η^2^); ^¥^ Within-group comparison; ^†^ Between-group comparison. Descriptive statistics are presented as mean ± standard deviation. Bold values indicate statistical significance (*p* < 0.05).

**Table 3:** Comparison of Corrected ICEB (ICEBc) Measurements Before and After Antihypertensive Treatment in Patients Presenting with Hypertensive Urgency: Discharged vs. Hospitalized Patients.

	Subgroups		Test Statistics ^†^		
	Discharged	Admitted	*F*	*p*	*η* ^2^
**ICEBc**					
Pre-treatment	5.26 ± 0.93	5.81 ± 0.81	2.133	0.151	0.043
Post-treatment	5.35 ± 0.81	5.61 ± 0.69	0.630	0.431	0.013
**Test Statistics ^¥^**	*F* = 1.148; *p* = 0.289; *η*^2^ = 0.023	*F* = 0.725; *p* = 0.399; *η*^2^ = 0.015			

F: Mixed Design ANOVA; Effect Size (η^2^); ^¥^ Within-group comparison; ^†^ Between-group comparison. Descriptive statistics are presented as mean ± standard deviation.

## Data Availability

The data presented in this study are available on request from the corresponding author due to privacy.

## Abbreviations

The following abbreviations are used in this manuscript:

ED	Emergency department
ICEB	Index of Cardiac Electrophysiological Balance
ICEBc	Corrected Index of Cardiac Electrophysiological Balance
ECG	Electrocardiography
QTc	Corrected QT interval

## References

[1] Afşin A., Asoğlu R., Kobat M., Asoğlu E., Suner A. (2020). Evaluation of index of cardio-electrophysiological balance in patients with atrial fibrillation on antiarrhythmic-drug therapy. Cardiol. Res..

[2] Astarita A., Covella M., Vallelonga F., Cesareo M., Totaro S., Ventre L., Aprà F., Veglio F., Milan A. (2020). Hypertensive emergencies and urgencies in emergency departments: A systematic review and meta-analysis. J. Hypertens..

[3] Chen X., Wang Z., Liu L., Zhang W., Tang Z., Liu B., Zhang X., Wei N., Wang J., Liu F. (2023). Prognostic value of index of cardiac electrophysiological balance among US middle-aged adults. Front. Cardiovasc. Med..

[4] Gauer R. (2017). Severe asymptomatic hypertension: Evaluation and treatment. Am. Fam. Physician.

[5] Çoksevim M., Kertmen Ö., Yıldırım U., Türkmen E. (2025). Ferritin and cardiac electrophysiology in end-stage renal disease: Evaluating the impact of index of cardio-electrophysiological balance. Hitit Med. J..

[6] Li F., Zhen Z., Sun S.-J., Jiang Y., Liang W.-H., Belau M., Storz R., Liao S.-Y., Tse H.-F. (2022). Attenuation of Myocardial Dysfunction in Hypertensive Cardiomyopathy Using Non-R-Wave-Synchronized Cardiac Shock Wave Therapy. Int. J. Mol. Sci..

[7] Tse G., Yan B.P. (2017). Traditional and novel electrocardiographic conduction and repolarization markers of sudden cardiac death. Europace.

[8] Henein M., Vancheri S., Longo G., Vancheri F. (2022). The impact of mental stress on cardiovascular health—Part II. J. Clin. Med..

[9] Karamanlıoğlu M., Şahan E. (2022). The relationship between white coat hypertension and the index of cardiac electrophysiological balance (ICEB). J. Health Sci. Med..

[10] Kulkarni K., Pallares-Lupon N., Arunachalam S., Bernus O., Walton R. (2023). Abstract 11632: Altered autonomic balance linked to arrhythmogenicity in chronic myocardial infarction ovine model. Circulation.

[11] Lin Y., Zhou F., Wang X., Guo Y., Chen W. (2023). Effect of the index of cardiac electrophysiological balance on major adverse cardiovascular events in patients with diabetes complicated with coronary heart disease. PeerJ.

[12] Liu Q., Yuan X., Sheng C., Cai W., Geng X., Liu H., Song S. (2024). Effect of long-term use of antipsychotics on the ventricular repolarization index. BMC Psychiatry.

[13] Yücetaş Ş., Kaya H., Kafadar S., Kafadar H., Tibilli H., Akçay A. (2022). Evaluation of index of cardiac-electrophysiological balance in patients with subarachnoid hemorrhage. BMC Cardiovasc. Disord..

[14] Lu H.R., Yan G.X., Gallacher D.J. (2013). A New Biomarker-index of Cardiac Electrophysiological Balance (iCEB)-Plays an Important role in Drug-induced Cardiac Arrhythmias: Beyond QT-prolongation and Torsades de Pointes (TdPs). J. Pharmacol. Toxicol. Methods.

[15] Cha S.A. (2022). Heart rate-corrected QT interval prolongation is associated with decreased heart rate variability in patients with type 2 diabetes. Medicine.

[16] Polatkan S.A.V., Gunay-Polatkan S., Isik O., Sigirli D. (2025). Using the Cardiac–Electrophysiological Balance Index to Predict Arrhythmia Risk After Colonoscopy. Medicina.

[17] Huang Y., Huang W., Mai W., Cai X., An D., Liu Z., Huang H., Zeng J., Hu Y., Xu D. (2017). White-coat hypertension is a risk factor for cardiovascular diseases and total mortality. J. Hypertens..

[18] Robyns T., Lu H.R., Gallacher D.J., Garweg C., Ector J., Willems R., Janssens S., Nuyens D. (2016). Evaluation of index of cardio-electrophysiological balance (iCEB) as a new biomarker for the identification of patients at increased arrhythmic risk. Ann. Noninvasive Electrocardiol..

[19] Solntseva T., Denisova A., Sivakova O., Chazova I. (2022). State of target organs damage and prevalence of clinical associated conditions in patients with a hypertensive crisis and uncontrolled hypertension (pilot study). J. Hypertens..

[20] Ozturk U., Ozturk O. (2020). Relation between index of cardio-electrophysiological balance and stroke severity in patients with acute ischemic stroke. Niger. J. Clin. Pract..

[21] Whelton P.K., Carey R.M., Aronow W.S., Casey D.E., Collins K.J., Dennison Himmelfarb C., DePalma S.M., Gidding S., Jamerson K.A., Jones D.W. (2018). 2017 ACC/AHA/AAPA/ABC/ACPM/AGS/APhA/ASH/ASPC/NMA/PCNA guideline for the prevention, detection, evaluation, and management of high blood pressure in adults: Executive summary. Hypertension.

[22] Williams B., Mancia G., Spiering W., Agabiti Rosei E., Azizi M., Burnier M., Clement D.L., Coca A., de Simone G., Dominiczak A. (2018). 2018 ESC/ESH guidelines for the management of arterial hypertension: The Task Force for the management of arterial hypertension of the European Society of Cardiology (ESC) and the European Society of Hypertension (ESH). Eur. Heart J..

[23] Yu Y., Wen S., Ruan Y., Liu N., Hu S., Duan X., Bai R. (2022). Impact of heart rate and rhythm on corrected QT interval during paroxysmal atrial fibrillation. Am. J. Cardiol..

[24] Rezuş C., Moga V.D., Ouatu A., Floria M. (2015). QT interval variations and mortality risk: Is there any relationship?. Anatol. J. Cardiol..

